# Induction of Neutralizing Antibodies against Four Serotypes of Dengue Viruses by MixBiEDIII, a Tetravalent Dengue Vaccine

**DOI:** 10.1371/journal.pone.0086573

**Published:** 2014-01-21

**Authors:** Hui Zhao, Tao Jiang, Xi-Zhen Zhou, Yong-Qiang Deng, Xiao-Feng Li, Shui-Ping Chen, Shun-Ya Zhu, Xi Zhou, E-De Qin, Cheng-Feng Qin

**Affiliations:** 1 Department of Virology, State Key Laboratory of Pathogen and Biosecurity, Beijing Institute of Microbiology and Epidemiology, Beijing, China; 2 Department of Life Science, Lanzhou University, Lanzhou, China; 3 State Key Laboratory of Virology, Wuhan University, Wuhan, China; University of Melbourne, Australia

## Abstract

The worldwide expansion of four serotypes of dengue virus (DENV) poses great risk to global public health. Several vaccine candidates are under development. However, none is yet available for humans. In the present study, a novel strategy to produce tetravalent DENV vaccine based on envelope protein domain III (EDIII) was proposed. Tandem EDIIIs of two serotypes (type 1–2 and type 3–4) of DENV connected by a Gly-Ser linker ((Gly_4_Ser)_3_) were expressed in *E. coli*, respectively. Then, the two bivalent recombinant EDIIIs were equally mixed to form the tetravalent vaccine candidate MixBiEDIII, and used to immunize BALB/c mice. The results showed that specific IgG and neutralizing antibodies against all four serotypes of DENV were successfully induced in the MixBiEDIII employing Freund adjuvant immunized mice. Furthermore, in the suckling mouse model, sera from mice immunized with MixBiEDIII provided significant protection against four serotypes of DENV challenge. Our data demonstrated that MixBiEDIII, as a novel form of subunit vaccine candidates, might have the potential to be further developed as a tetravalent dengue vaccine in the near future.

## Introduction

Dengue fever (DF) and dengue hemorrhagic fever (DHF) are acute febrile diseases transmitted by mosquitoes, posing an increasing public health threat globally. About 2.5 billion people, two fifths of the world's population, are now at risk of infection and 50 million cases of DF were reported worldwide every year [1]. The disease has now expanded in more than 100 countries in recent decades [2]. However, there is no effective dengue vaccine yet available for humans, although efforts have been made for over 60 years. Current vaccine candidates under development include live-attenuated, live-recombinant and inactivated viruses, as well as subunit vaccines based on recombinant proteins or naked DNA constructs, among which some vaccine candidates are progressing in clinical trials [3]–[6], but a licensed vaccine will not be available in a few years [7], [8].

DENV contains four serotypes, and each of them can cause a wide spectrum of clinical manifestations, including mild DF, severe DHF and deadly dengue shock syndrome [9]. Infection with one DENV serotype provides life-long protective immunity against that serotype, not the other serotypes. Currently, the pathogenesis of severe diseases remains poorly understood, and secondary infection with another DENV serotype is theoretically believed to increase the risk of severe diseases via the mechanism of antibody dependent enhancement (ADE) in Fcγ receptor-bearing cells [10]–[13]. Hence, an ideal DENV vaccine should induce tetravalent, balanced, protective antibody response to the four serotypes while minimizing the risk of enhancement [14]–[16].

The conventional tetravalent DENV vaccines based on live-attenuated DENVs have not resulted in overall efficacy during the clinical trials, and suggested that viral interference is a possible factor contributing to its low efficacy [5], [8]. For economic reasons, an inactivated vaccine became a potentially less attractive candidate for use in DENV-endemic areas than other vaccine candidates [17]. Thus, attention is increasingly being focused on subunit strategies [18].

DENV contains a 10.7 Kb RNA genome encoding three structural proteins (C, prM and E), and seven nonstructural proteins (NS1, NS2A, NS2B, NS3, NS4A, NS4B, and NS5). Neutralizing antibodies are largely induced by the E protein which is divided into three domains (EDI, EDII and EDIII) based on the X-ray crystal structures [19], [20]. The most potent neutralizing antibodies against DENV bind to EDIII and have been shown in some cases to be effective as passive prophylaxis in rodents [21]–[23]. In contrast, the role of antibodies to EDI/EDII tends to be more cross-reactive and less potent in neutralization [24], [25]. Multiple serotype-specific neutralizing epitopes of the E protein have been mapped within EDIII of DENV [21], [26], [27], and anti-EDIII antibodies are recognized as the most powerful blockers of viral infectivity [21]. Importantly, compared with EDI/EDII, EDIII has a low potential for inducing cross-reactive antibodies to heterologous DENV, which might be implicated in the pathogenesis of DHF [28]. Thus, EDIII, rather than full-length E protein, has emerged as a promising region for a subunit vaccine candidate.

Several works have been reported that EDIII can be expressed in various expression system, including *E. coli*, *Pichia pastoris* yeast, and insect cells, and induce specific immunity responses against DENV in mice or non-human primates [29]–[33]. Recent years, to enhance the immunological features and immune responses of EDIII, some approaches have been developed, including virus-like particles display, combined an immunomodulator and virus replicon particles [34]–[36]. However, only a clinical trial with a monovalent formulation of the DEN1-80E component was conducted with preliminary results suggesting that the vaccine was safe and immunogenic DENV [14].

In this study, a novel kind of tetravalent DENV vaccine was produced based on the mixture of two bivalent EDIIIs of DENV in *E. coli*. This tetravalent vaccine, named MixBiEDIII, was evidenced to evoke humoral immune responses and protective antibodies against all four serotypes DENV.

## Results

### Characterization of the Two Tandem Bivalent Recombinant DENV EDIIIs

**Figure 1:**
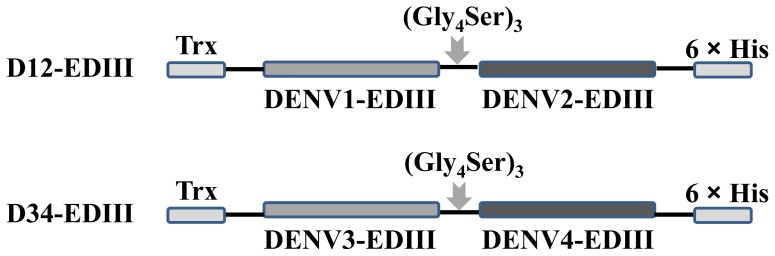
Schematic representation of tandem EDIII constructs. The amino acid sequence covering EDIII (298aa∼400aa) from each serotype was arranged sequentially, and (Gly_4_Ser)_3_ sequence was used to connect EDIII regions. Thioredoxin (Trx) was fused at the N-terminal to increase solubility and the C-terminal His-Patch was included for purificaiton through Ni-NTA agarose.

**Figure 2:**
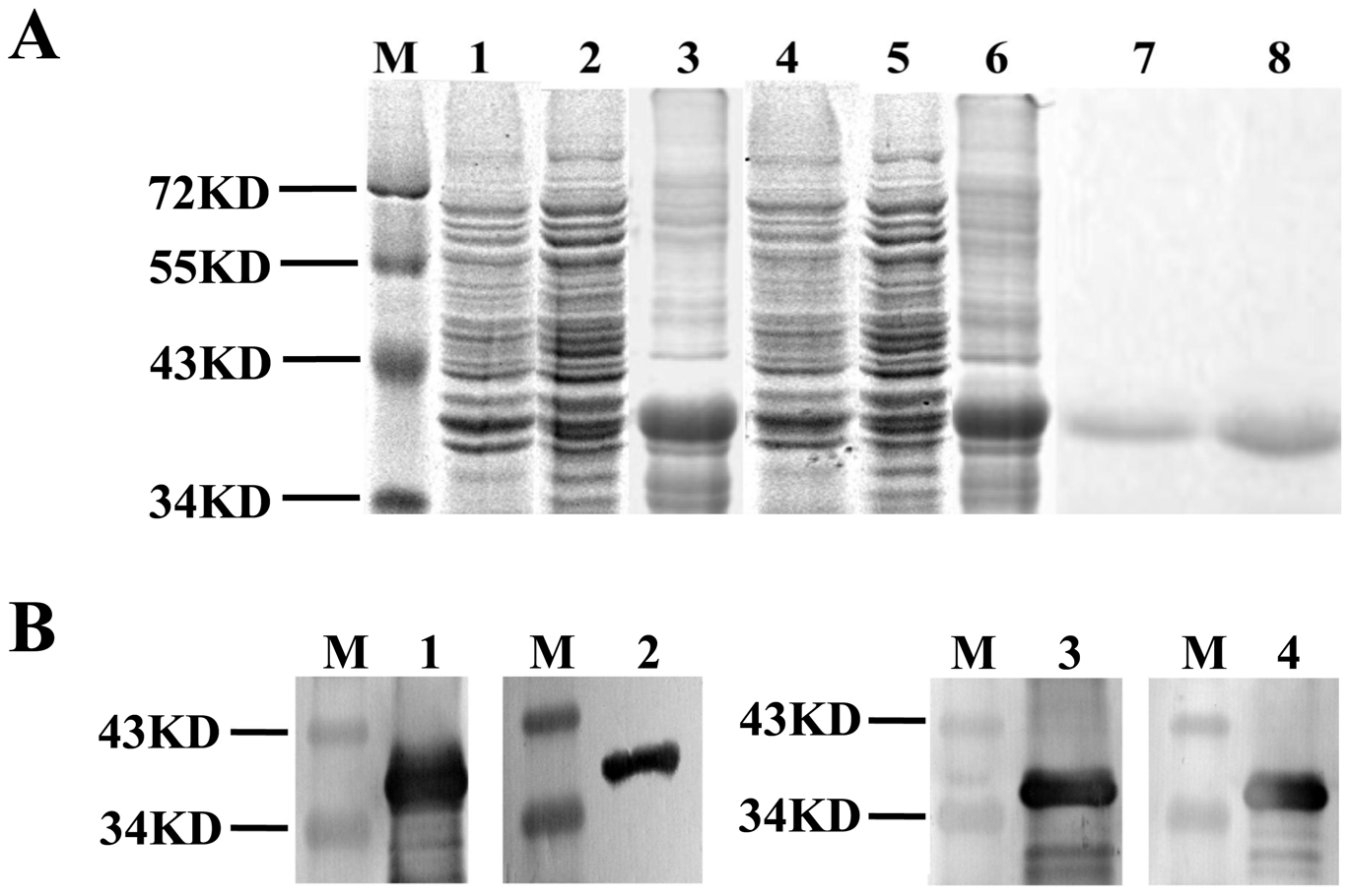
Expression and purification of the tandem bivalent recombinant EDIII antigens. (A) Coomaissee staining of SDS-PAGE with samples of expression and purification of the tandem bivalent recombinant EDIIIs, including lysates from uninduced and arabinose-induced *E. coli* cells, and Ni-NTA agarose purified fractions was shown. Prominent protein bands of about 40 KD were visible in both induced and purified fractions. Lanes 1 and 4: lysates from uninduced *E. coli* (receiving pBAD-D12-EDIII and pBAD-D34-EDIII plasmid, respectively). Lanes 2 and 5: the supernatant from arabinose induced *E. coli* lysate after sonication. Lanes 3 and 6: the pellet from lysate. Lanes 7 and 8: the eluate (D12-EDIII and D34-EDIII protein, respectively) purified through Ni-NTA agarose. Lane M: protein marker. (B) Western blotting analysis of purified EDIII proteins by monoclonal antibody against DENV1-4, respectively. Lanes 1 and 2, the recombinant D12-EDIII protein is recognized by the corresponding monoclonal antibody, respectively. Lanes 3 and 4, the recombinant D34-EDIII protein is recognized by the corresponding monoclonal antibody, respectively. Lane M: protein marker.

To produce the tandem bivalent recombinant DENV EDIII, the EDIIIs of two serotypes of DENV were sequentially connected by a (Gly_4_Ser)_3_ linker (Fig. 1). Thioredoxin (Trx) was fused at the N-terminal to enhance solubility and the C-terminal His-Patch was included for purification on metal-chelating resin. The expression plasmid pBAD-D12-EDIII and pBAD-D34-EDIII was transformed into *E. coli* for the tandem bivalent EDIII expression, respectively. The induction profile of a typical *E. coli* clone harboring expression plasmid is shown in Fig. 2A. The induction of arabinose lead to the appearance of a new approximately 40 KD band consisted with the predicted size of the tandem bivalent EDIII (D12-EDIII or D34-EDIII). The majority of recombinant protein remained in the pellet (lane 3 and 6) and very little in the supernatant (lane 2 and 5) after sonication. Hence, the expressed recombinant proteins were mainly in the form of inclusion bodies in *E. coli*. After purification through Ni-NTA agarose, the solution containing the recombinant protein D12-EDIII or D34-EDIII was isolated as a single band (lane 7 and 8). Furthermore, western blotting assays showed that the tandem bivalent EDIIIs could be detected by corresponding monoclonal antibodies, respectively, and suggested that recombinant D12-EDIII and D34-EDIII protein was successfully produced (Fig. 2B).

**Figure 3:**
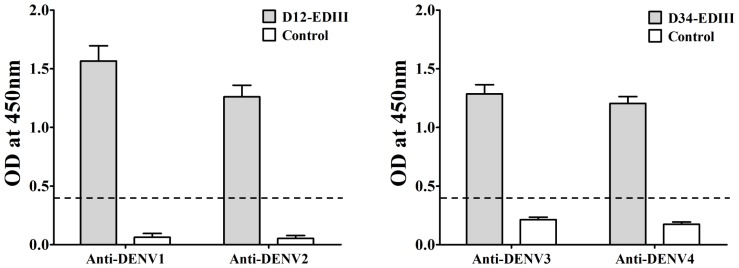
Characterization of the tandem bivalent recombinant EDIII antigens. ELISA of purified EDIII proteins was performed using corresponding monoclonal antibody against different serotype of DENV. Trx protein was control. The cut-off for the ELISA is shown by a dotted line.

To detect the antigenicity of the purified recombinant D12-EDIII and D34-EDIII protein, ELISA with anti-DENV monoclonal antibodies were performed. The results showed that the tandem bivalent EDIIIs strongly reacted with corresponding monoclonal antibodies, respectively (Fig. 3), and the fusion protein Trx failed to be recognized by DENV antibodies, which demonstrated that the tandem bivalent EDIIIs were potential to be DENV antigen as originally designed.

### MixBiEDIII Induced Neutralizing Antibodies against Four Serotypes of DENVs

**Figure 4:**
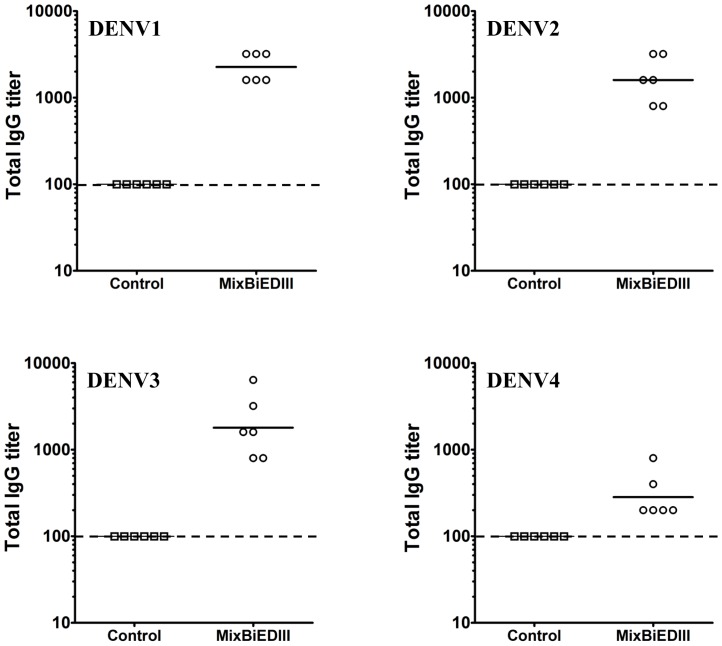
IgG antibody responses in mice immunized with MixBiEDIII. The total lgG titers of immune sera from mice immunized with MixBiEDIII at two weeks after the last immunization were measured by ELISA. The lgG titers of immune sera against four DENV serotypes from mice immunized with MixBiEDIII were significant higher than control group. Dotted line represents limits of detection.

**Figure 5:**
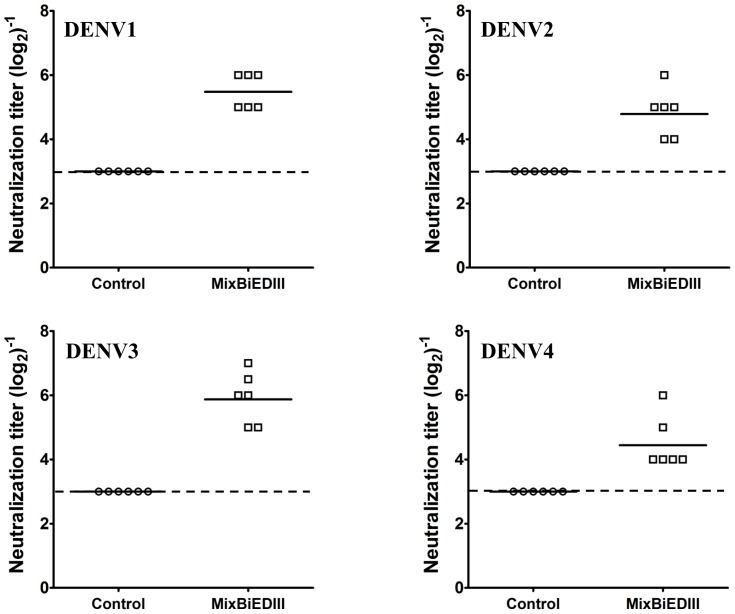
Neutralizing antibody responses in mice immunized with MixBiEDIII. Neutralization titers of MixBiEDIII immune sera at two weeks after the last immunization were measured by PRNT_50_. The neutralizing antibodies against four DENV serotypes were significantly induced by MixBiEDIII compared with control group. Dotted line represents limits of detection.

The tetravalent vaccine, MixBiEDIII, was prepared by 1∶1 mixture of D12-EDIII and D34-EDIII. Then, group of BALB/c mice was immunized with MixBiEDIII three times at two-week intervals. Group of mice immunized with Trx was set as control. Serum samples were collected at two weeks after the last boost, and serotype specific IgG antibody titers of sera were assayed by ELISA using heat inactivated DENV as the capture antigen. The results revealed that IgG antibodies against four serotypes of DENV were induced in MixBiEDIII-immunized mice (Fig. 4), and Trx-immunization failed to induce DENV antibodies as expected. Neutralizing antibodies against four serotypes of DENV were then assayed by 50% plaque reduction neutralization test (PRNT_50_) (Fig. 5). Neutralizing antibodies against four DENV serotypes were all induced by MixBiEDIII, and the geometric mean titers were 1∶45, 1∶29, 1∶57 and 1∶22 against DENV1 to 4, respectively. Mice immunized with Trx failed to induce DENV-specific neutralizing antibodies as expected (<1∶8). Taken together, these results demonstrated immunization with MixBiEDIII induced humoral immune responses including specific IgG and neutralizing antibodies against all four serotypes of DENV in mice.

### MixBiEDIII Provided Partial Protection against Lethal DENVs Challenge

**Figure 6:**
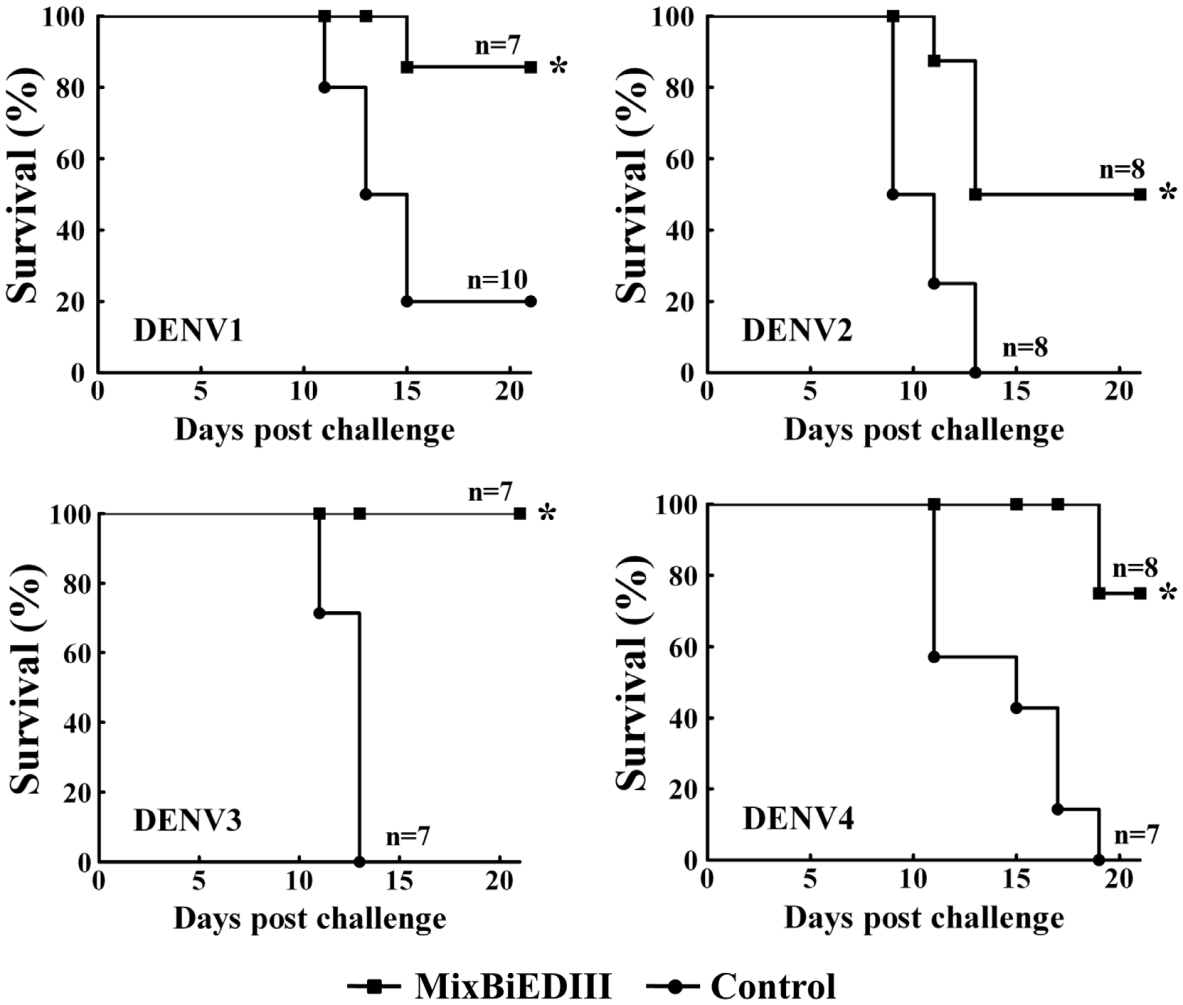
Protection of anti-MixBiEDIII sera against all four DENV serotypes in suckling mice. Sera from mice immunized with MixBiEDIII were pre-incubated with different serotype DENV and inoculated into suckling mouse (1-day-old) brain intracranially (IC), and mortality was recorder. (*) Survival rate of mice inoculated with virus plus sera from MixBiEDIII group was statistically significant compared with control group using Fisher’s exact test.

Finally, the protective efficacy of tetravalent MixBiEDIII antigen was assessed in a suckling mouse model as described previously [37], [38]. Two weeks after the last boost, sera from mice immunized with MixBiEDIII or with Trx were used to determine the presence of protective antibodies. As shown in Fig. 6, the sera from MixBiEDIII immunized mice was capable of providing protection from any one of the DENV1 to 4 infection, and complete protection against DENV3 was observed. The level of neutralization *in vivo* against all four DENV serotypes was statistically significant compared with the corresponding control groups (*p* = 0.013, 0.038, 0.00029 and 0.006, respectively). In addition, MixBiEDIII immunization significantly prolonged the mean survival time compared with the control by Log-Rank analysis (Table 1). Taken together, these data showed that MixBiEDIII immunization could be partial protective in mice against all four serotypes of DENVs challenge.

**Table 1:** Protection of anti-MixBiEDIII sera against all four serotypes DENV.

Sera group	Mean survival days			
	DENV1	DENV2	DENV3	DENV4
**MixBiEDIII**	19.8±1.1^a^	16.8±1.5^a^	21±0^a^	20.5±0.31^a^
**Control**	14.8±1.1	10.5±0.6	12.7±0.3	14.4±1.3

The suckling mice were injected intracranially (IC) with the sera-virus mixture, and mortality was monitored for 21 days. Data represent the mean±SD.

a P<0.05 for MixBiEDIII sera group vs. control sera group, by Log-Rank analysis.

## Discussion

Different approaches have been used to obtain the tetravalent vaccine based on EDIII, including mixture of four monovalent EDIII, a consensus EDIII of the four serotypes and the tandem tetravalent EDIII [18], [30], [37], [39]–[43]. In the present work, to develop a tetravalent DENV vaccine, we firstly constructed the tandem EDIII of two serotypes of DENV. The bivalent antigens were created by connecting the EDIIIs of two serotypes of DENV by a flexible linker. Here, a (Gly_4_Ser)_3_ polypeptide linker, which is widely used in antibody engineering, was used to connect the two EDIIIs so that the bivalent EDIIIs could retain their structural integrity without being subjected to any constraints at the fusion junction. Glycine lacks of a beta carbon and serine is of propensity for hydrogen bonding, thus Gly-Ser linker are preferred for tandem protein expression [44]. Khanam et al. also developed a construct containing the EDIIIs of DENV2 and DENV4 linked by a Gly-Ser linker [45].

Immunization with MixBiEDIII in mice induced IgG and neutralizing antibodies against all four serotypes of DENV. The EDIII-specific IgG antibody response in mice appears somewhat divergent among different DENV serotypes, with relative low titer against DENV4. Such DENV4 EDIII immunogenic inferiority has also been observed by others [39], [42], [46], and remains unexplained. The results presented here indicate that some antigenic determinants are perhaps buried inside, especially those in EDIII of DENV4 and DENV2. Hence, a more suitable linker should be further improved so that most antigenic determinants of MixBiEDIII can be exposed outside. Recently, a tetravalent mixture of insect cell-secreted recombinant EDIIIs was reported to elicit PRNT_50_ titers of 1∶1,196, 1∶3,174, 1∶378, and 1∶254 against DENV1, DENV2, DENV3, and DENV4, respectively [39]. In contrast, the EDIII-based plasmid mixture was about 1∶10 for each of the four DENV serotypes [38]. These differences are likely a reflection of the nature of antigen, route of immunization, and differences in the experimental parameters of the PRNT_50_ assay [40]–[42]. Whatever, our data clearly demonstrated that the capability of MixBiEDIII to elicit neutralizing, and therefore, presumably protective antibodies against all four DENV serotypes.

In absence of a good mouse model for DENV infection [47], suckling mouse model has been widely used to evaluate protective efficacy of dengue vaccines [38], [48], [49]. Our data showed that sera from MixBiEDIII-immunized mice significantly neutralized the lethal dose of four serotypes of DENV in sucking mouse, which suggested immunization using MixBiEDIII is potential to provide protection to mouse against the challenge of all four serotypes of DENV.

Only partial protection was observed except for DENV3 in the model, which was in association with the relatively lower neutralizing antibodies. To improve antibody responses and achieve complete protective immunity, we will test recombinant EDIIIs in combination with a plasmid vector encoding the same antigen in a recombinant protein prime–plasmid boost strategy in the future. DNA vaccine can efficiently elicit humoral and cellular immune responses, and have preponderances in simple preparation and low cost. Several approaches based on EDIII have also focused on DNA [3], [38], [50], [51]. Recently, Azevedo et al. demonstrated that combined immunization with a DNA vaccine and chimeric live attenuated vaccine leads to synergistic protective effect against a lethal dose of DENV2, when compared to each vaccine administered alone [52]. Additionally, It was reported that the cellular immune response played an important role in protecting against DENV in the mouse model and suggested that a safe and efficient vaccine against dengue should trigger both humoral and cellular immunity [53], [54]. Cellular immune responses induced by EDIII recombinant protein expressed in *E. coli* were also observed by Babu et al [55]. Thus, further studies should be required to test T cell responses in MixBiEDIII-immunized mice.

Previously, it has been thought that antibodies to prM or EDI/II are highly cross-reactive among the DENV serotypes and, even at high concentrations, do not neutralize infection but potently promote ADE [28], [56]. Thus, dengue vaccine candidates based on EDIII eliminate the anti-prM or EDI/II antibodies, and might lower the risk of ADE. However, according to the recent reports, only the quaternary epitopes expressed on the intact virion but not on soluble EDIII could induce protective neutralizing antibodies in human [57]–[60]. Li et al. also found that some monoclonal antibodies (mAbs) induced by EDIII were inefficient to neutralization potency, because of the epitopes targeted by these mAbs are not exposed on the virion surface, which suggested that EDIII might mainly elicited a poorly neutralizing, cross-reactive antibody response [61]. However, data from White’s report demonstrated that the E antigen induced only serotype-specific neutralizing antibodies, which predominantly targeted EDIII [62]. In the future, cross-serotype reactive responses induced by the each bivalent EDIII construct should be determined to well understand the antibody response elicited by a tetravalent dengue vaccine based on bivalent EDIIIs, which may offer insights into DENV vaccine development.

In conclusion, tetravalent vaccine MixBiEDIII could elicit protective neutralizing antibodies against all four serotypes of DENV challenge. These results have important implications for DENV subunit vaccine based on EDIII.

## Materials and Methods

### Cells, Viruses, and Antibodies

The mosquito C6/36 cells were grown in RPMI-1640 medium (Invitrogen) containing 10% fetal bovine serum (FBS) (Hyclone, Logan, UT) at 28°C [63]. Baby hamster kidney BHK-21 cells were maintained at 37°C in DMEM medium (Invitrogen) containing 10% FBS [63]. The stocks of DENV1 strain GZ/80 (GenBank accession no. AF350498), DENV2 strain 43 (GenBank accession no. AF204178), DENV3 strain 80-2 (GenBank accession no. AF317645), and DENV4 strain B5 (GenBank accession no. AF289029) were propagated in C6/36 cells and titered in BHK-21 cells using standard plaque forming assay. Four serotype-specific mouse monoclonal antibodies were prepared in our lab and used in this study.

### PCR Amplification and Plasmid Construction

The cDNA fragments of tandem EDIII (corresponding to amino acids residues 298 to 400 of the E protein of DENV) of two serotypes(DENV1 and DENV2, DENV3 and DENV4)were amplified by PCR using LA Taq DNA polymerase (TaKaRa) and recombinant plasmid pBAD-B1234 [20] as template with the following conditions: initial denaturation at 94°C for 2 min; 30 cycles at 94°C for 30 s, 55°C for 30 s, and 72°C for 45 s; and a final extension at 72°C for 7 min. The following oligonucleotide primers 5′-TCA TAT GTG ATG TGC ACG GGC TCA TTC AAG C-3′ and 5′-TTG GCC GAT AGA ACT TCC TTT CTT AAA CCA GT-3′ were used to amplify tandem EDIII of DENV1 and DENV2 (D12-EDIII), and 5′-AGC TAT GCA ATG TGC TTG AAT ACC TTT GTG T-3′ and 5′-CTT GCC AAT GGA ACT CCC TTT CCT GAA CCA AT-3′ for tandem EDIII of DENV3 and DENV4 (D34-EDIII). The PCR products were purified with a Qiaquick Gel extraction kit (Qiagen) and ligated into the pBAD/Topo ThioFusion vector (Invitrogen) to generate the recombinant plasmid pBAD-D12-EDIII and pBAD-D34-EDIII, respectively. The recombinant plasmids were finally confirmed by restriction enzyme digestion and DNA sequencing.

### Protein Expression and Purification

The plasmids pBAD-D12-EDIII and pBAD-D34-EDIII were transformed in TOP10 cells using the One Shot TOP10 Chemically Competent *E. coli* kit (Invitrogen), respectively. The transformed TOP10 cells were then cultured overnight and induced by 0.002% arabinose (Invitrogen) at 37°C. The transformed cells were harvested by centrifugation, and the inclusion bodies were solubilized with 8 M urea. The recombinant proteins were then purified through Ni-NTA agarose (Invitrogen) as previously described [64]. Followed by dialysis in phosphate buffered saline (PBS) and the concentration of D12-EDIII and D34-EDIII were determined by bicinchoninic acid (BCA) Protein Assay Kit (Pierce), respectively.

### SDS-PAGE and Western Blotting Assay

Protein samples were mixed with loading buffer (0.1 M Tris-HCl (pH 8.8), 20% glycerol, 1% DTT, and 3% SDS and 0.0025% bromphenol blue), and loaded onto a homogeneous 12% polyacrylamide gel. Western blotting analysis was performed using anti-DENV monoclonal antibodies described as above. In brief, the PVDF membranes were incubated with antibody against four serotypes at room temperature for 1 h, followed by three washes in TBST (0.1% Tween 20/Tris-buffered saline). An alkaline phosphatase-conjugated goat anti-mouse IgG at a dilution of 1/1000 was used to detect the primary antibody. Membrane blots were developed with BCIP/NBT phosphatase substrate (KPL).

### ELISA

The ELISA plate was coated with 100 ng of D12-EDIII proteins or D34-EDIII proteins (100 µl/well) overnight in 0.05 M carbonate buffer, pH 9.6. After incubation with 150 µl of 5% fat-free milk in PBS in each well for 1 h at 37°C to prevent nonspecific binding, each of 1∶100 diluted anti-DENV monoclonal antibodies (100 µl/well) was added to corresponding well and incubated for 1 h at 37°C. Trx protein (Sigma) was used as control. Then anti-mouse IgG antibodies conjugated to horseradish peroxidase were added to wells (100 µl/well) and incubated for 30 min at 37°C. Finally, the plate was incubated in dark with 100 µl/well of TMB One solution (Promega) at 37°C for 15 min. The reaction was stopped by addition of 2 M H_2_SO_4_ and the absorbance was read at 450 nm in an ELISA reader (Beckman). The cut-off in the ELISA is set at 0.4.

### Mice Immunization

Groups of 4-week-old female BALB/c mice (n = 6) were inoculated with 100 µg of tetravalent mixture (containing 50 µg of D12-EDIII and D34-EDIII, respectively) (MixBiEDIII group), or Trx protein (control group) by subcutaneous route with Freund’s complete adjuvant (Sigma), respectively. All mice were then boosted twice with the same dose in Freund’s incomplete adjuvant (Sigma) at an interval of 2 weeks. Two weeks after each immunization, serum samples were prepared and stored frozen until use.

### IgG Antibody Detection

The IgG antibody in the sera from immunized mice was detected by ELISA. In brief, the plate was coated with heat inactivated DENV overnight at 4°C. All sera samples serially diluted (two fold) in PBS were incubated in triplicate wells (100 µl/well) for 1 h at 37°C. Following procedures were made as described in Methods “ELISA”. IgG antibody titers were expressed by the highest serum dilution at which the absorbance at 450 nm is higher than 0.4.

### Neutralization Assay

Sera from immunized mice were assayed for neutralizing antibodies against DENV by PRNT_50_ as previously described [63]. Briefly, all serum samples (heat inactivated) were used to prepare two fold serial dilution starting at 1∶8. Diluted sera were mixed 1∶1 with DENV suspension containing 100 plaque forming units (PFU) and incubated for 1 h at 37°C. Then, the sera-virus mixture was added to monolayer BHK-21 cells in a 6-well plate in duplicate and incubated for another 1 h at 37°C. Following this, the supernatant was removed, and 1 ml of 1.0% (w/v) LMP agarose (Promega) in DMEM plus 2% (v/v) FBS was layered onto the infected cells. After further incubation at 37°C for 4 to 7 days, the wells were stained with 1% (w/v) crystal violet dissolved in 4% (v/v) formaldehyde to visualize the plaques. The percentage of plaque reduction was calculated as previously described [65].

### Neutralizing Assay *in vivo*

Sera from immunized mice were assayed for protecting antibodies against DENV as previously described [20]. At first, the 50% lethal dose (LD_50_) for each serotype of DENV was determined in groups of 1-day-old BALB/c mice. Each serotype of DENV (50 LD_50_) was 1∶1 mixed with pooled sera from the immunized mice. The sera-virus mixture (volume 30 µl) was then incubated for 1 h at 37°C and injected intracranially (IC) into suckling mice. Mice were then observed daily and mortality was recorded.

### Statistical Analysis

For the neutralizing assay *in vivo* experiments, the surviving rate was evaluated using Fisher’s exact test. Mean survival time was compared using Log-rank analysis using OriginPro software v8.0.

### Ethics Statements

All animal experimental procedures were carried out in strict accordance with and approved by the Animal Experiment Committee of Beijing Institute of Microbiology and Epidemiology, Beijing, China.
